# Causes of Hospital Violence, Characteristics of Perpetrators, and Prevention and Control Measures: A Case Analysis of 341 Serious Hospital Violence Incidents in China

**DOI:** 10.3389/fpubh.2021.783137

**Published:** 2022-01-07

**Authors:** Yuanshuo Ma, Licheng Wang, Yongchen Wang, Zhe Li, Yafeng Zhang, Lihua Fan, Xin Ni

**Affiliations:** ^1^Department of Health Management, School of Health Management, Harbin Medical University, Harbin, China; ^2^Department of General Practice, The Second Affiliated Hospital of Harbin Medical University, Harbin, China; ^3^Beijing Children's Hospital, Capital Medical University, National Center for Children's Health, Beijing, China

**Keywords:** hospital violence, healthcare workers, perpetrators, prevention and control measures, China, characteristics and measures

## Abstract

**Objective:** Hospital violence remains a global public health problem. This study aims to analyze serious hospital violence causes in China and the characteristics of perpetrators. It likewise seeks to understand frontline personnel's needs and put forward targeted suggestions.

**Methods:** Serious hospital violence cases from 2011 to 2020 in the China Judgment Online System (CJOS) were selected for descriptive statistical analysis. A total of 72 doctors, nurses, hospital managers, and security personnel from 20 secondary and tertiary hospitals in China were selected for semi-structured interviews.

**Results:** Of the incidents, 62.17% were caused by patients' deaths and dissatisfaction with their treatment results. Moreover, it was found that out-of-hospital disputes (11.14%) were also one of the main reasons for serious hospital violence. The perpetrators were mainly males (80.3%), and had attained junior high school education or lower (86.5%). Furthermore, most of them were family members of the patients (76.1%). Healthcare workers urgently hope that relevant parties will take new measures in terms of legislation, security, and dispute handling capacity.

**Conclusion:** In the past 10 years, serious hospital violence's frequency in China has remained high. Furthermore, their harmful consequences are more serious. The causes of hospital violence are diverse, and the characteristics of perpetrators are obvious. Frontline healthcare workers urgently need relevant parties to take effective measures in terms of legislation, security, and dispute handling capacity, to prevent the occurrence of violence and protect medical personnel's safety.

## Introduction

Hospital violence can be defined as an incident in which healthcare workers or providers are abused, threatened, or attacked in connection with their work (1). It involves an explicit or implicit threat healthcare workers' safety, well-being, or health (1). Hospital violence exists in many countries in the world. As such, it is still a global public health problem (2). According to the World Health Organization (WHO), about 8–38% of healthcare workers suffered from physical violence at work in 2019 (3). This figure is even higher in Asia (4). This is why workplace violence in China's hospitals has been the focus of many previous studies. Since 2020, the killing of doctors in the emergency department of Beijing Civil Aviation General Hospital (5, 6) and the explosion of the First Affiliated Hospital of Zhejiang University (7) have pushed the vicious violence problem to the forefront of public opinion in China.

There have been many previous studies on hospital violence. A nationwide survey of healthcare workers in China showed that hospital violence incidence was at 65.8%. Verbal violence accounted for 64.9% of the total hospital violence cases, while physical violence and sexual harassment accounted for 11.8 and 3.9%, respectively (8). Workplace violence incidence among healthcare workers in children's hospitals in China is more serious than that in general hospitals. A total of 68.6% of healthcare workers in children's hospitals in China have experienced at least one hospital violence incident (9). Patient factors such as emotional control ability, mental state, education level, and gender may be risk factors for hospital violence. Similarly, medical factors such as work experience, service system efficiency, and healthcare workers' poor communication abilities may be risk factors as well (10–12). Hospital violence's continuous occurrence has had a serious negative impact on healthcare workers' physical and mental health. This resulted in some healthcare workers having negative emotions such as anxiety (13–15), depression (16, 17), job burnout (18), and job dissatisfaction (18). Furthermore, it even directly or indirectly caused medical personnel to have suicidal tendencies. Hospital violence also seriously affects the doctor–patient relationship and makes doctors practice defensive medical behavior. This reduces the quality of health services and makes the trust between doctors and patients extremely fragile. Therefore, the contradiction between doctors and patients is further deepened (19–22). In the long run, hospital violence is bound to have a far-reaching negative impact on China's medical and health system.

In the past few years, the Chinese government has recognized hospital violence's negative impact on China's doctor–patient relationship and the development of China's health cause. Relevant laws were promulgated and a series of measures were taken, but hospital violence still occurs frequently. Thus, it still poses a serious threat to healthcare workers' physical and mental health and their order of diagnosis and treatment in the hospital. This phenomenon further increases the estrangement between doctors and patients, worsening the already fragile doctor–patient relationship (23). It has become the common expectation of all sectors of society to take more targeted measures in effectively preventing and controlling hospital violence.

There have been relatively few previous studies on actual cases of serious hospital violence. The data from these studies came from network reports. Thus, the accuracy and comprehensiveness of the information were poor, making it difficult to reflect the real situation of serious hospital violence in China. The present study will use Chinese court judgments as research data. It will extract real and accurate information related to hospital violence systematically, and comprehensively analyze the factors inducing hospital violence, and explore perpetrators' characteristics. This will provide a basis for relevant parties to aid in preventing and controlling the occurrence of hospital violence. At the same time, based on the analysis of inducing factors and relevant characteristics, this study will select medical personnel and managers of hospitals at all levels for in-depth interviews. This will help us understand frontline personnel's urgent needs regarding the prevention and control of hospital violence. This, in turn, will lead to more targeted and effective prevention and control measures.

## Methods

### Sample and Data Collection

This study's qualitative data comes from the judgment documents published in the China Judgment Online System (CJOS), which is operated and maintained by the Supreme People's Court of China. The case judgments of all courts in China's 31 provinces can be retrieved from this website (except for special cases involving national security, juvenile delinquency, and criminal crimes, or cases people's courts should not publish on the Internet). We used January 1, 2011 to December 31, 2020 as the search dates to study the occurrence of serious hospital violence in China. We used keywords such as “hospital,” “violence,” “hospital violence,” “medical dispute,” “hospital order,” “violent injury doctor,” “doctor,” “nurse,” and “healthcare workers” for single keyword retrieval or combined keyword retrieval. We were able to retrieve 63,262 judgments from our searches. Four graduate students carefully read and screened these judgments, and we excluded repeated cases and those that had nothing to do with hospital violence. For judgments with missing relevant information, we searched the relevant information of the events on Baidu news, Weibo, Netease News, Tencent News, Sina News, and other Internet platforms according to the events' time, place, and other information. The events' missing information were supplemented through a mutual confirmation of news information from multiple websites. After excluding irrelevant judgments, we were left with 341 judgments which involved a total of 873 violent criminals.

Based on the above data, we conducted expert consultation and subject group discussion, and determined the outline of a semi-structured interview. The outline consisted of two parts: basic information and open-ended questions. Basic information included five questions regarding gender, age, education, occupation, and hospital level of the interviewees. There were two open-ended questions (Appendix 1). Using this interview outline, we conducted semi-structured interviews with convenient sampling of 72 doctors, nurses, hospital managers, and security personnel from 20 secondary and tertiary hospitals in China from March 2021 to June 2021. This study sample was chosen as they have a deeper understanding of hospital violence and more experience in preventing such incidents. To avoid bias in the interview results due to different levels of hospitals, our survey included secondary and tertiary hospitals. To a certain extent, Beijing and Heilongjiang Province can represent the current situation of China's economically developed and underdeveloped provinces, respectively. Therefore, we selected medical personnel in medical institutions in these two provinces to ensure that interviews reflect frontline medical personnel's actual need to prevent and control hospital violence. Furthermore, interviews help put forward targeted and effective countermeasures to prevent and control hospital violence.

The researchers explained the study's purpose to the respondents and obtained their informed consent before the interview. The researchers conducted face-to-face interviews in an independent place, with Chinese as the interview language. The entire interview process was recorded. The interview began with an open-ended question: “Can you describe a violent incident in the hospital workplace that impacted you most.” The follow-up question was “Based on your personal experience, what measures must be taken to effectively prevent and control such incidents?” Each interview lasted 32–71 min and was conducted during the respondents' free time. A HKUST iFLYTEK Voice Recorder was used to record each interview. HKUST iFLYTEK voice recorder is an intelligent recording device, which can automatically identify the use scene, reduce noise, and safely store the recorded content in the cloud. The respondents' real names were replaced by pseudonyms to ensure their anonymity. After the interview, the recordings were transcribed verbatim.

### Variable Coding and Data Analysis

After the screening of judgments, five researchers carefully read 50 judgments and selected relevant characteristic variables of hospital violence. They then conducted group discussions according to their selection results to determine the variables' standard names, types, and specific codes. A total of 18 characteristic variables related to violence were identified. The research team divided these variables into two categories: serious hospital violence incidents and perpetrators. Nine basic information variables (hospital level, department, cause, means of implementation, category of victim, and consequences of violence) were identified regarding serious hospital violence incidents. Similarly, nine characteristic variables (gender, age, education, occupation, criminal record, history of violent crime, mental state, and relationship with patients) were identified for the perpetrators. The five researchers then read 341 judgments in detail according to the selected variables and picked the variable information. The researchers conducted descriptive statistical analysis on the characteristics of hospital violence. All statistical analyses were performed using SPSS 25.0 and Microsoft Excel 2019.

Content analysis is a research method that subjectively explains text data's content through the systematic classification process of coding and identifying topics or patterns. It is used to analyze open problem data (24). In this study, two social medicine doctoral students with qualitative research experience independently coded the data using hybrid inductive and deductive coding methods (24). The inconsistency between the students' codes was solved through a panel discussion to complete the interview materials' coding. The coding of interview data was carried out using the NVivo 12 software.

## Results

### Basic Information on Severe Hospital Violence

**Figure 1:**
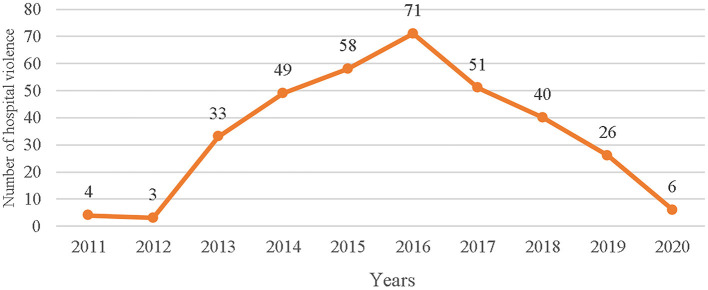
The frequency of serious workplace violence (WPV) between 2011 and 2020.

From 2011 to 2020, Chinese courts ruled on a total of 341 hospital violence incidents. China's serious hospital violence showed a trend of first increase and then decrease in the past 10 years (Figure 1). Most of these hospital violence incidents occurred in secondary hospitals (54.3%), outpatient departments (47.8%), and emergency departments (25.2%). Doctors and nurses experienced the highest hospital violence frequency. Most perpetrators commit violence by physically assaulting others, laying wreaths, blocking doors, burning papers, placed the corpse, and pulling banners, among others. The largest number of incidents resulted in hospital property losses, disorders, and minor injuries to medical personnel.

### Causes of Hospital Violence

**Figure 2:**
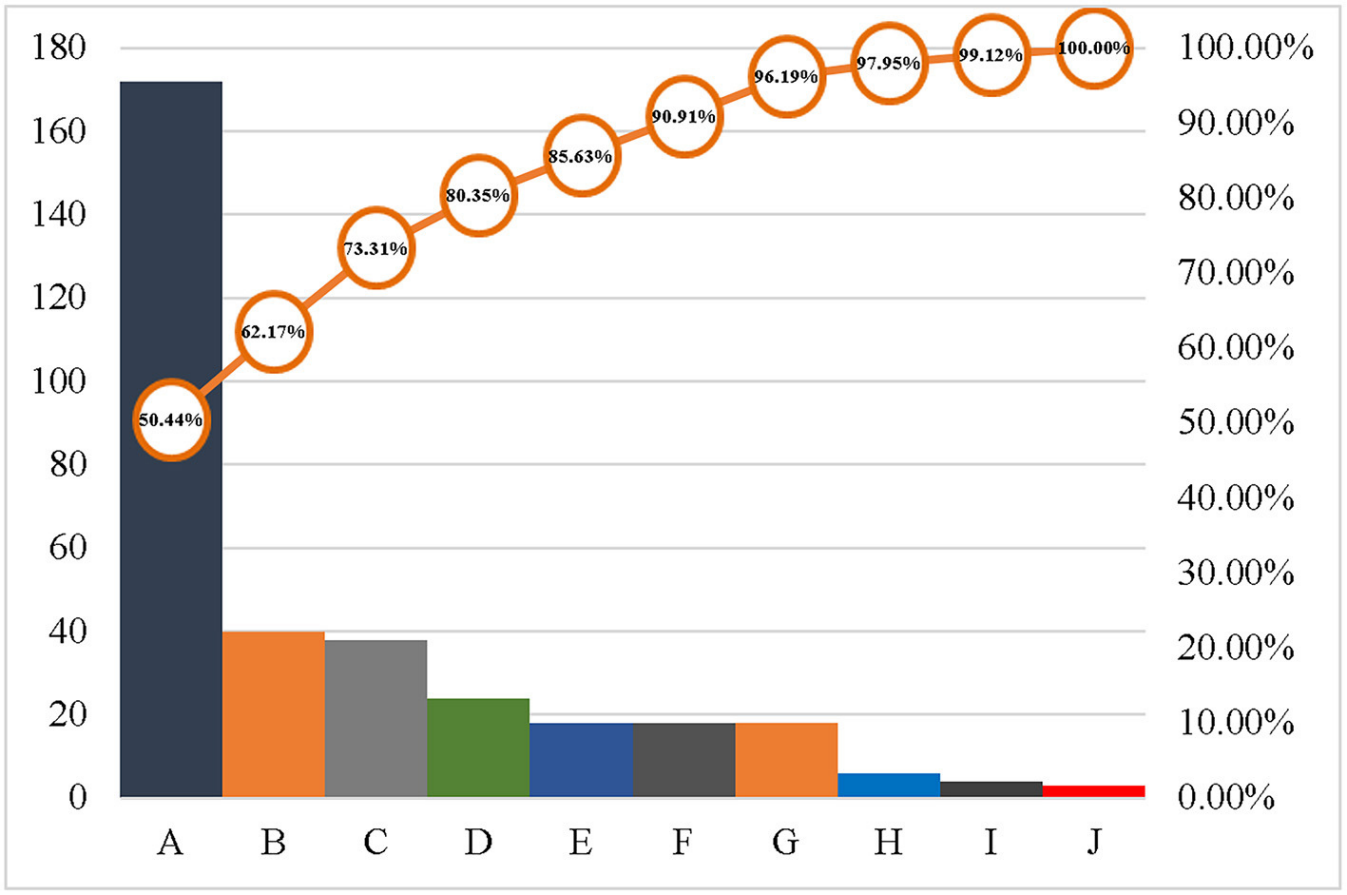
Causes of hospital violence.

Note: A: Patient died; B: Dissatisfied with the effect of treatment; C: Out-of-hospital disputes; D: Dissatisfied with healthcare workers arrangements; E: The patient's unreasonable request was rejected; F: Medical Dispute; G: Attitudes of medical staff; H: other; I: waiting time; J: Medical expenses.

The results showed that the main causes of hospital violence were patients' death (50.44%), dissatisfaction with the treatment effect (11.73%), out-of-hospital disputes (11.14%), and dissatisfaction with the arrangement of healthcare workers (7.04%). In contrast, waiting time and medical expenses were only secondary factors (Figure 2).

### Characteristics of Perpetrators

Through an analysis of the perpetrators' characteristics, the researchers found that a vast majority of the 873 perpetrators were men (80.3%), under the age of 40 (58.0%), had attained junior middle school education or below (86.5%), and were farmers or unemployed (85.2%). Seventy-two people had criminal records, of which 45 had a history of violent crimes. The main perpetrators were patients' family members (76.1%) (Table 1).

**Table 1:** Characteristics of perpetrators.

**Variables**	**Total**	
	***n***	**Percentage (%)**
**Gender**		
Male	701	80.3
Female	172	19.7
**Age**		
≤ 20	20	2.3
21–40	486	55.7
41–60	326	37.3
≥61	41	4.7
**Education level**		
Illiteracy	59	6.8
Primary school	393	45.0
Junior high school	303	34.7
High school	102	11.7
≥College degree	16	1.8
**Occupation**		
Unemployed	246	28.2
Farmer	410	47.0
Individual	18	2.1
Civil servants	13	1.5
Business owners	9	1.0
Retire	9	1.0
Worker	134	15.3
Enterprise employee	22	2.5
Freelancer	7	0.8
Other	5	0.6
**Criminal history**		
Yes	72	8.2
No	801	91.8
**History of violent crime**		
Yes	45	5.2
No	828	94.8
**Mental state at the time of violence**		
Sober	810	92.8
Drunk	56	6.4
Mental disorder	7	0.8
**Relationship with patients**		
Patient	82	9.4
Relatives of patients	664	76.1
Patient friend	8	0.9
Persons who have disputes with patients	100	11.5
Patient's hometown	19	2.2

### Hospital Violence Prevention and Control Measures

A total of 72 people were interviewed, 33 from secondary hospitals and 39 from tertiary hospitals. Most of them were over 35 years old (55.55%) and had a Bachelor's degree (54.17%). Particularly, there were 16 doctors, 22 nurses, 24 managers, and 10 security guards. The interview results show that frontline medical personnel believe that hospital violence's effective prevention and control at this stage should be carried out from three different aspects. First, the state should make a special legislation to increase the punishment and cost of malignant and violent medical injuries. At the same time, there must be publicity and education for ordinary people to improve their health and legal literacy. Second, hospitals should constantly improve their doctors' diagnoses, treatment, and security ability. Moreover, they must improve their medical dispute-handling team's abilities and that of their dispute-handling personnel. Third, hospitals should train their medical personnel in doctor–patient communication, risk identification abilities, and knowledge and skills. Healthcare workers should also take the initiative to improve their skills to further avoid the occurrence of hospital violence (Table 2).

**Table 2:** Interview analysis on prevention and control measures of hospital violence.

**Theme**	**Subtopics**	**Quotes (e.g.)**
Strengthening legal construction	Special legislation	“At present, there is no special law to punish the acts of wounding and killing doctors, which makes our safety not guaranteed by law. Moreover, the hospital is a public place, and the existing law does not define the hospital as a public place. Therefore, I think the existing law is not enough for the prevention and control of hospital violence.”
	Punishment intensity	“The existing laws have relatively little punishment for the perpetrators of violence. The noisy behavior of some personnel in the hospital and the injury behavior of medical personnel cannot be punished. As a result, people follow this behavior one after the other without fear.”
Increased security	Number of security personnel	“I think the number of security personnel in our hospital is too small. As a result, the security personnel in some departments cannot arrive at the place of the incident in time and cannot effectively stop the occurrence of violence in time.”
	Quality and stability of security team	“At present, the security personnel of the hospital cooperates with the security company. The security personnel is not employed by the hospital. The salary of the security personnel is low, resulting in their greater mobility. Moreover, most of the security personnel are over 55 years old, and they are older, so they cannot play a deterrent role to the vicious violent medical criminals.”
	Security equipment	“We hope that the government can provide us with some equipment to prevent hospital violence. These include installing one-button alarm devices, installing high-quality monitoring equipment, and providing anti-riot tools for security personnel. The shortage of existing hardware leads to the hospital's inability to deal with malignant violent medical incidents.”
Improve the ability of medical personnel to prevent risks	communication skills	“The communication ability of medical personnel is very important for the good or bad doctor–patient relationship. As long as the communication ability of medical personnel is strong, they can also appease the perpetrators who may have violent medical injuries and avoid the occurrence of violent medical injuries. A good doctor should not only have superb medical skills, but also have superb doctor–patient communication skills.”
	Risk identification ability	“Through my daily observation, I found that healthcare workers who often work long hours suffer less hospital violence, because they can find some signs and signs of violence from patients or their families,. They can likewise resolve this risk according to their own work experience. This is a skill worth learning by every healthcare worker.”
	Personal technical level	“We recognize that the causes of violence are both patient factors and medical factors. Sometimes, due to the limited technical level of medical personnel, the patient's condition worsens, causing damage to the patient to a certain extent, which will lead to the violence of the patient or the patient's family members.”
Improve the procedure and environment	Hospital environment	“China has a large population, resulting in a large number of patients in Chinese hospitals. However, they only have a few healthcare workers, resulting in an imbalance of the proportion of doctors and patients. Our hospital is an old hospital area with a narrow space. The patients treated every day have been crowded, and there is not even a temporary rest place. It is difficult to keep up with all aspects of hardware facilities, which will lead to dissatisfaction with the hospital before meeting the doctor.”
	Hospital treatment process	“When many patients or their family members come to the hospital for treatment, because they are not familiar with the treatment process of the hospital, they repeatedly run around and fail to complete registration, treatment, payment, medicine collection and other matters, which is easy to cause patients' dissatisfaction with the hospital or healthcare workers, resulting in violent medical injuries.”
Improve the hospital's dispute resolution capacity	Dispute settlement system	“Some hospitals do not have a complete set of standardized guidelines for dispute handling, so there are no rules to follow in dealing with medical disputes, which is chaotic. The handling scales of disputes are different, which is very easy to further worsen the contradiction between doctors and patients.”
	Dispute-handling ability	“The ability of dispute-handling personnel is very important for the settlement of disputes. Some people can solve the events smoothly, while others can't. In addition, the professional composition of the members of the dispute-handling team is also very important, especially in law, psychology, and clinic. Only when all majors understand it can it be conducive to the resolution of the incident.”
Strengthen publicity and education for the general public	Publicity and education	“Many patients or their families believe that when they come to the hospital for diagnosis and treatment, the doctor must cure the patient's disease and the patient will not deteriorate. However, this situation is obviously due to the lack of medical knowledge of patients or their families, and the expectation of diagnosis and treatment results is unreasonable. Therefore, it is necessary for the government, hospitals, and even medical personnel to strengthen health education for patients and their families, to make their expectations return to a reasonable range.”

## Discussion

The characteristics of hospital violence events found in this study are consistent with those of previous studies. Most of these cases occurred in secondary hospitals, outpatient departments, and emergency departments (25, 26). Moreover, most perpetrators committed violence by placing wreaths, blocking doors, burning papers, placed the corpse, and physically assaulting others (26, 27). Doctors and nurses experienced the highest frequency of violence (28, 29). The incidents' consequences were mainly minor injuries, property damage, and disorderly disturbances (30, 31). This study will focus on the causes of hospital violence and the characteristics of the perpetrators. It will likewise explore possible hospital violence prevention and control measures to alleviate hospital violence in the future, and provide reference and advice for governments and policy-making bodies involved in creating such measures.

### Analysis of the Causes of Hospital Violence

Through an analysis of the causes of violence, we found that patients' deaths and dissatisfaction with treatment effects were the main causes of serious hospital violence. This may be caused by patients' or their families' high expectations regarding the effects of diagnosis and treatment (23, 32). Patients often choose hospitals with a good environment and a high expectation regarding treatment effects. Once the treatment effect is poor, the high expectation brought about by the good environment before the treatment and the low perceived value of patients after the treatment will lead to lower patient satisfaction. This will result in the occurrence of hospital violence (33). However, we should also clearly realize that patients' deaths and poor treatment effects may likewise be caused by medical accidents or errors. It is difficult for patients or their families to accept the damage caused to patients. This too, can easily cause hospital violence.

We also found another main reason for serious hospital violence that has never been mentioned in previous studies: out-of-hospital disputes. The results show that out-of-hospital disputes ranked third among serious hospital violence's main causes. Out-of-hospital disputes refer to disputes between patients and third-party personnel (i.e., non-medical personnel) before admission. Such a dispute may have caused damage to the patient's physical and mental health and may have led to the patient's admission. After the patient is admitted to the hospital, the altercation between the two parties is likely transferred to the hospital or healthcare workers, if it is not reasonably resolved. Thus, the hospital becomes a place for patients to take out their negative emotions. Moreover, such emotions are likely vented out on medical personnel. Hospital managers and healthcare workers should always be vigilant during such cases to avoid the transfer and evolution of contradictions.

Dissatisfaction with healthcare workers' arrangements and the failure to meet patients' unreasonable requirements are also main reasons for the occurrence of hospital violence. In the process of seeing a doctor, patients sometimes refuse to listen to healthcare workers' arrangements or put forward unreasonable requirements due to their own needs. Rejection of unreasonable requirements and environmental factors such as long waiting times, increase the likelihood of patients to engage in violent actions to hurt healthcare workers to express their dissatisfaction.

Medical disputes are doctor–patient contradictions caused by patients' disagreement with the diagnosis and treatment process or results. When the doctors and patients have inconsistent negotiations on the disputes, patients may often recourse to violence to express their dissatisfaction with the entire healthcare system, or with specific doctors (32). Moreover, due to the influence of some media on doctors' “stigmatization” and previous violent medical incidents' success, patients or their families tend to imitate others in committing violence to achieve their own goals.

Dissatisfaction with healthcare workers' attitudes is also a main cause of hospital violence. Healthcare workers' attitude problems is in the final analysis of communication problems between doctors and patients. At present, the government and hospitals are carrying out only a few doctor–patient communication trainings for healthcare workers. Moreover, there is a lack of human resources, resulting in the heavy medical tasks undertaken by medical personnel every day. As such, there is a lack of opportunity and sufficient time to receive relevant training. This may result in healthcare workers' insufficient humanistic care for patients and lack of empathy. This will further lead to poor effects of doctor–patient communication. When the patient believes that healthcare workers have a poor attitude toward them, hospital violence may occur (23). Research has proven that strengthening humanistic medical education and improving healthcare workers' humanistic medical literacy can effectively promote trust between doctors and patients. This will likewise reduce the conflict between doctors and patients (34).

Long waiting times and high medical expenses have always been important reasons behind violence committed by patients. However, we believe that the hospital violence caused by long waiting times and high medical expenses will gradually decrease with the increasing investment of the Chinese government in the health sector and the continuous improvement of the medical insurance system and the hierarchical diagnosis and treatment system.

### Analysis of the Characteristics of Perpetrators

Through a study of the relevant characteristics of 873 perpetrators, it was found that most of the perpetrators were male, 21–40 years old, with junior high school level education or below, and were farmers or unemployed. This finding consistent with previous research results (35, 36). Patients or their families with such characteristics have a low level of overall health literacy and often have high expectations from medical procedures or treatment results. They are more inclined to take extreme violence to vent their negative emotions and exert pressure on the hospital when their expectations or requirements are not met. Furthermore, according to the neutralization theory, “the attraction of higher loyalty obligation is one of the factors that neutralizes internal control and external control and makes the offender embark on the road of misconduct. The perpetrators claim that their actions are in line with the moral obligations of their group, leading to the ineffectiveness of internal control (i.e., self-factors restraining criminal acts) and external control (i.e., social factors restraining criminal acts)” (37). Therefore, people with low educational backgrounds and poor cognitive levels may have a higher sense of identity for group behavior. Similarly, they are more likely to be coerced by the group to resort to violent injury behavior.

The results show that some perpetrators have criminal records and a history of violent crimes. Based on previous studies, it is generally believed that a history of violent crimes is the main factor in predicting patients' or their families' hospital behavior (38–40). Therefore, medical personnel should pay attention to personnel with such characteristics in the process of preventing and controlling hospital violence. However, because the police system is not connected to the hospital system, it is difficult for healthcare workers to judge whether the patient or their family has a criminal record.

The study also found that drunkenness or mental disorders may also be characteristics of violent perpetrators in hospitals. This is consistent with previous research results (36, 40). When patients or their families are not satisfied with the work of healthcare workers or the hospital's diagnosis and treatment process, those in a state of drunkenness or having mental disorders are more impulsive and difficult to control. This results to their violent acts toward healthcare workers.

We also found that most perpetrators were patients' relatives, further confirming previous research (26). We believe that this is caused by a lack of empathy of patients' relatives toward the doctor. Such an inducement lies in their in-group identity. As a social primate, it is instinctual for a man to rely on his group for survival. Under the control of this instinct, people stay close to their group, and trust those in the same group to help and protect each other. However, there is relative indifference to other groups they do not belong to. Moreover, they may be hostile toward individuals or groups that infringe on the interests of their own group. In cases of hospital violence, patients' relatives often consider healthcare workers as parties infringing on their group interests just because their expectations are not fully met. As such, they do not empathize with these healthcare workers and even resort to perpetrating violent injury against them. Healthcare workers also have the same problem. The lack of empathy among healthcare workers leads them to ignore the demands of patients. This results in the creation and escalation of doctor–patient contradictions, leading to further occurrences of hospital violence.

### Analysis of Hospital Violence Prevention and Control Measures

The harm caused by hospital violence to hospitals, healthcare workers, and the national health system cannot be ignored. All concerned parties should continue to pay attention to this problem and take active measures to curb the occurrence of violent medical injuries. This study puts forward the following specific measures based on the analysis of the characteristics of serious hospital violence incidents, in-depth interviews with frontline health workers and managers, and the actual situation of hospital violence prevention and control in China. These measures will help provide a reference for the effective prevention and control of hospital violence.

### Develop a Hospital Workplace Violence Risk Reporting System

Davide Ferorelli evaluated the incident reporting system for clinical risk management and found that the system can effectively reduce litigation between doctors and patients (41). At the same time, the scholar also proposed corresponding clinical risk management methods to measure the risk of psychiatric violence (42). Hospital violence also requires clinical risk management. The occurrence of such incidents is not without warning. There must be some omens and signs before the incident. Hospital managers and researchers should develop and improve the risk reporting system of hospital violence by summarizing the causes and precursors of hospital violence in the future. The corresponding risk prevention tools help to timely identify the risk of violent incidents and take targeted preventive measures to avoid such incidents.

### Continuing to Reform the Medical and Health System and Increasing Investments in the Health Sector

The occurrence of hospital violence partly reflects the disadvantages of China's medical and health systems. For example, inadequate health system investments result in insufficient training costs for medical personnel. Moreover, it becomes difficult to pay expensive remuneration for their services. This leads to medical malpractice, corruption, poor communication between doctors and patients, and hospital violence (43). Therefore, the government should increase investments in the health sector and improve the quality of medical services. Through systematic reform, the government and hospitals can ease the dispute between doctors and patients and reduce the occurrence of hospital violence (27).

### Improving the Legal System Construction Against Hospital Violence

The law is an effective means of regulating violence. Although China has successively issued relevant laws and regulations for dealing with hospital violence (6), the punishment for perpetrators is light, and the effect of deterring criminals from committing crimes is limited. Thus, using the other countries' experience as a basis, China should make special legislations on hospital violence. They must also distinguish the punishment for hospital violence from those for other forms of general violence and impose stricter punishments. This will help China achieve the purpose of warning and regulation.

### Strengthening the Publicity and Education of the General Public's Concept of the Health and Legal System

The gradual enhancement of patients' awareness of their rights and improving their understanding of medical services' particularity will exacerbate the conflict between doctors and patients. The state should pay attention to the popularization and education of people's basic health and medical knowledge through media publicity and community education. Through this, patients will have a clearer and reasonable understanding of their diseases and will be able to more easily accept the adverse effects of such diseases (44). At the same time, legal education for the masses should be strengthened to reduce their criminal motives.

### Strengthening the Security Force of the Hospital

Security guards are an important force for maintaining law and order in the hospital. However, Chinese hospitals' security are generally weak. Security personnel's characteristics such as old age, poor treatment, and high mobility result in their ineffective response to security threats. They may even choose to escape when violence occurs in the hospital. Therefore, a younger security team and improvements in security personnel's treatment and overall quality are crucial for Chinese hospitals to prevent and control workplace violence. At the same time, using digital information in response to security forces' deficiencies is also an effective measure that the hospital can adopt during instances of hospital violence. Strengthening hospital security capacity is crucial in preventing the occurrence of violence after effectively identifying their characteristics.

### Improving the Dispute-Handling System and the Dispute-Handling Ability of Personnel

Personnel's personal ability in dispute handling, the hospital dispute-handling team's abilities, and the national medical dispute-handling system's establishment and improvement are crucial to avoid the occurrence of hospital violence (27). The improvement of the dispute-handling system and personnel's dispute-handling abilities can effectively prevent the occurrence of violent medical injuries caused by the failure of doctor–patient negotiations on controversial issues.

### Improving the Hospital Treatment Process and Environment

Patients' awareness of their rights is being gradually strengthened and their requirements for medical experience are increasing with society's development. The hospital treatment environment and the treatment process' complexity directly affect the patients' intuitive feelings toward hospitals. Previous studies have shown that improvements in the hospital environment, such as setting clear department instruction signs, creating a clean and comfortable environment, and implementing a convenient medical treatment process can improve patients' medical experience. These will improve patient satisfaction and reduce the likelihood of disputes (40). Therefore, in the process of hospital management, hospital managers should constantly optimize the hospital's diagnosis and treatment process and create a harmonious and orderly treatment environment. They must likewise avoid affecting patients' medical experience and healthcare personnel's working mood brought about by the complexity and noise of the diagnosis and treatment process, which can result in hospital violence.

### Improving the Self-Protection Awareness and Ability of Healthcare Workers

Training can effectively prevent hospital violence occurrence (45). Hospitals should provide various forms of training to healthcare workers. These include training and lectures to improve medical personnel's doctor–patient communication abilities, hospital violence risk identification and response abilities, and medical technology levels. At the same time, emergency drills should be actively carried out to prevent and control hospital violence. This will improve medical personnel's prevention and control awareness. Medical personnel should also actively study hard, participate in trainings, and apply the learned skills to practice. They can effectively protect themselves from violence by identifying the risk of violence according to the characteristics of violent events and perpetrators, taking the initiative to avoid danger, and taking preventive measures.

## Limitations

This study had three limitations. First, Due to the delay in court judgment, some violent medical incidents that have occurred have still not been judged by the court. Thus, they were not included in the scope of the study. Second, the occurrence of serious violent medical trauma may be a result of a joint action of multiple factors. The variables involved in this study were limited. As such, some confounding factors were not considered. Therefore, further studies are required in the future. Third, the selected interviewees included only the hospitals' relevant personnel. In the future, government personnel and university researchers should be included in the research to achieve more comprehensive and systematic prevention and control measures. Lastly, convenience sampling is not suitable for general inference.

## Conclusions

The frequency of serious hospital violence events in China is still high. Hospital violence is mostly caused by patient death, dissatisfaction with treatment, and out-of-hospital disputes. These disputes are closely related to the gender, age, education, occupation, and other characteristics of the perpetrator. All concerned parties should take new measures from the aspects of legislation, security, dispute-handling systems, and capacity building to prevent and control the occurrence of hospital violence. Medical personnel should also improve their protection awareness and risk prevention ability. Furthermore, they must take advanced preventive and control measures according to patients' characteristics to protect themselves from violence.

## Data Availability Statement

The data analyzed in this study is subject to the following licenses/restrictions: If necessary, the data can be obtained by contacting the corresponding author. Requests to access these datasets should be directed to Lihua Fan, lihuafan@126.com.

## Ethics Statement

The studies involving human participants were reviewed and approved by the Ethics Committee of the School of Public Health of Harbin Medical University (Project Identify Code: HMUIRB20180305). Written informed consent from the participants' legal guardian/next of kin was not required to participate in this study in accordance with the national legislation and the institutional requirements.

## Author Contributions

YM participated in study design and conception, data acquisition, data analysis, manuscript drafting, and funding acquisition. LW and YW participated in data acquisition. ZL and YZ participated in the design and conceptualization of the study, acquisition of data, and data interpretation. LF and XN participated in the design and conceptualization of study, acquisition of data, revising of the manuscript, acquisition of funding, and supervision. All authors were involved in the manuscript's revision and approved this final version.

## Funding

This research was funded by the National Natural Science Foundation of China, Grant Number 71874043. The funders had no role in the design of the study and collection, analysis, interpretation of data, and in writing the manuscript.

## Conflict of Interest

The authors declare that the research was conducted in the absence of any commercial or financial relationships that could be construed as a potential conflict of interest.

## Publisher's Note

All claims expressed in this article are solely those of the authors and do not necessarily represent those of their affiliated organizations, or those of the publisher, the editors and the reviewers. Any product that may be evaluated in this article, or claim that may be made by its manufacturer, is not guaranteed or endorsed by the publisher.
