# Supplementary material for: Causes of Hospital Violence, Characteristics of Perpetrators, and Prevention and Control Measures: A Case Analysis of 341 Serious Hospital Violence Incidents in China
Source: Front Public Health. 2022 Jan 7;9:783137. doi: 10.3389/fpubh.2021.783137 (PMC8777225; doi:10.3389/fpubh.2021.783137)
Supplement: Supplementary file 1 [file Data_Sheet_1.docx]

Basic Information:

1. Gender：①Male ②Female

2. Age:

3. Education level: ①≤College degree and below, ②undergraduate, ③≥master

4. Profession： ①doctors, ②nurses, ③managers, ④security guards.

5. Hospital level：①secondary hospitals, ②tertiary hospitals

Questions:

1. Can you describe a violent incident in the hospital workplace that impacted you most？

2. Based on your personal experience, what measures must be taken to effectively prevent and control such incidents?
